# Gd-EOB-DTPA dynamic contrast-enhanced magnetic resonance imaging is more effective than enhanced 64-slice CT for the detection of small lesions in patients with hepatocellular carcinoma: Erratum

**DOI:** 10.1097/MD.0000000000025405

**Published:** 2021-04-02

In the article, “Gd-EOB-DTPA dynamic contrast-enhanced magnetic resonance imaging is more effective than enhanced 64-slice CT for the detection of small lesions in patients with hepatocellular carcinoma”^1^, the number of male patients listed in section 2.1. Patients appears incorrectly as 153 and should be 144 male patients.
